# Subsite-specific association of DEAD box RNA helicase DDX60 with the development and prognosis of oral squamous cell carcinoma

**DOI:** 10.18632/oncotarget.13197

**Published:** 2016-11-08

**Authors:** Ting-Ying Fu, Chao-Nan Wu, Huei-Cin Sie, Jiin-Tsuey Cheng, Yaoh-Shiang Lin, Huei-Han Liou, Yu-Kai Tseng, Chih-Wen Shu, Kuo-Wang Tsai, Leing-Ming Yen, Hui-Wen Tseng, Ching-Jiunn Tseng, Luo-Ping Ger, Pei-Feng Liu

**Affiliations:** ^1^ Department of Pathology and Laboratory Medicine, Kaohsiung Veterans General Hospital, Kaohsiung, Taiwan; ^2^ Department of Optometry, Shu-Zen Junior College of Medicine and Management, Kaohsiung, Taiwan; ^3^ Department of Stomatology, Kaohsiung Veterans General Hospital, Kaohsiung, Taiwan; ^4^ Department of Dental Technology, Shu-Zen Junior College of Medicine and Management, Kaohsiung, Taiwan; ^5^ Department of Biological Sciences, National Sun Yat-Sen University, Kaohsiung, Taiwan; ^6^ Department of Otorhinolaryngology-Head & Neck Surgery, Kaohsiung Veterans General Hospital, Kaohsiung, Taiwan; ^7^ Department of Medical Education and Research, Kaohsiung Veterans General Hospital, Kaohsiung, Taiwan; ^8^ Department of Orthopedics, Show Chwan Memorial Hospital, Changhua, Taiwan; ^9^ Department of Orthopedics, National Cheng Kung University Hospital, Tainan, Taiwan; ^10^ Department of Chemical Biology, National Pingtung University of Education, Pingtung, Taiwan; ^11^ Department of Dermatology, Kaohsiung Veterans General Hospital, Kaohsiung, Taiwan; ^12^ Department of Nursing, Meiho University, Pingtung, Taiwan; ^13^ Institute of Clinical Medicine, National Yang-Ming University, Taipei, Taiwan; ^14^ Department of Pharmacology, National Defense Medical Center, Taipei, Taiwan; ^15^ Department of Medical Research, China Medical University Hospital, China Medical University, Taichung, Taiwan; ^16^ Institute of Biomedical Sciences, National Sun Yat-Sen University, Kaohsiung, Taiwan; ^17^ Department of Biotechnology, Fooyin University, Kaohsiung, Taiwan

**Keywords:** DDX60, oral cancer, subsite-specific, tumorigenesis, prognosis

## Abstract

The clinical significance and biological function of DEXD/H box helicase 60 (DDX60) in oral cancer remains unknown. Herein, we evaluated the association of DDX60 expression with tumorigenesis and the prognosis of oral squamous cell carcinoma (OSCC). DDX60 expression was examined by immunohistochemistry on tissue microarray slides of 494 OSCC patients, including 180 buccal mucosal SCC (BMSCC), 241 tongue SCC (TSCC), and 73 lip SCC (LSCC) patients. DDX60 expression was significantly increased in all three subsites of OSCC compared to its expression in tumor adjacent normal tissues. However, its association with tumorigenesis was specific to the oral cavity subsite after the stratification of betel quid chewing, smoking, and drinking. Among OSCC patients, higher levels of DDX60 expression were associated with the male gender, a well-differentiated tumor, advanced stage of disease, and a large tumor size with subsite specific features. LSCC patients with high DDX60 expression levels showed shorter disease-specific survival, particularly those with moderately or poorly differentiated tumors. Additionally, TSCC or OSCC patients with high DDX60 expression showed a poor disease-free survival (DFS), particularly those with moderately or poorly differentiated tumors. Therefore, DDX60 is a novel and unfavorable biomarker for tumorigenesis and prognosis of OSCC in a subsite-specific manner.

## INTRODUCTION

DEAD-box (DDX) proteins, distinguished by the presence of a conserved amino-acid sequence Asp-Glu-Ala-Asp motif, are the largest family of RNA helicases, with 37 members in humans [1]. DDX proteins interact with RNAs including rRNAs and mRNAs to perform many normal cellular functions, such as translation initiation, mRNA synthesis, RNA splicing and modification, ribosome and spliceosome assembly, and the transcriptional regulation of the genes involved in DNA repair and proliferation, cell cycle arrest, and apoptosis, highlighting the potentially involvement of DDX proteins in cancer [2-10]. DDX60, a novel DEAD box RNA helicase, is induced after a virus infection. The helicase domain of DDX60 binds to viral RNA and DNA, and its ATP-binding site is essential for DDX60-activated RIG, leading to type I interferon (IFN) expression [11-13]. DDX60 also induces RIG-I-independent viral RNA degradation [11, 13]. DDX60 and its highly similar homolog DEAD box polypeptide 60-like (DDX60L) have recently been described as interferon-stimulated products upon a viral infection. However, DDX60L plays a distinct and specific function in restricting hepatitis C virus replication [14]. Although DDX60 is involved in protection against viral infections, the clinical significance and biological function of DDX60 in cancers, particularly oral cancer, remain largely unknown.

More than 90% of oral cancers are classified as oral squamous cell carcinoma (OSCC), typically observed on the tongue, buccal mucosa, and lips. The habitual use of substances [such as cigarette smoking, alcohol drinking, and betel quid (BQ) chewing] [15, 16], chronic periodontitis [10, 17], and viral infections [18, 19] are major risk factors for OSCC. The overall 5-year survival rate of OSCC patients has remained at approximately 50% for several decades [8, 20]. Moreover, local recurrence remains a major challenge in OSCC [21]. Thus, identifying reliable biomarkers to predict disease-specific survival (DSS) and the recurrence of OSCC is an emerging issue. To date, many studies have attempted to identify novel biomarkers for OSCC, such as fibronectin 1 (FN1) [22], integrin alpha4beta1 (ITGA4) [22], syndecan-2 (SDC2) [22], glycoprotein CD44 [22], AXL [23], matrix metalloproteinases (MMP) 1 and MMP10 [24]. However, many of these studies are limited to the gene expression or protein levels due to a small numbers of patients. Our preliminary next generation sequencing and real-time PCR data indicated that DDX60 gene expression was increased in OSCC tissues compared to that of the corresponding tumor adjacent normal tissues (CTAN) (data not shown), suggesting that DDX60 may be involved in the tumorigenesis of OSCC. In this study, we compared the level of DDX60 expression between tumor and CTAN tissues using immunohistochemistry (IHC) to assay tissue microarray slides constructed from 180 buccal mucosa SCC (BMSCC), 241 tongue SCC (TSCC), and 73 lip SCC (LSCC) patients. Then, the correlation between DDX60 expression and the patients' clinicopathologic features and survival was extensively evaluated.

## RESULTS

### The comparisons of DDX60 expression between tumor and CTAN tissues of BMSCC, TSCC, LSCC, and OSCC patients

**Figure 1:**
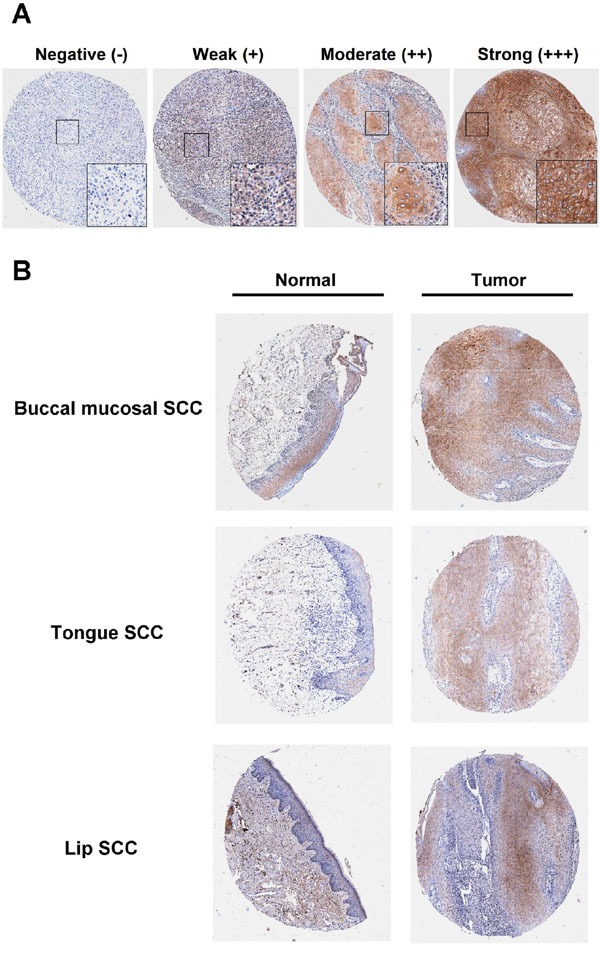
IHC staining of DDX60 protein expression **A.** The representative immunoreactivity intensity of DDX60 in OSCC for negative (-), weak (+), moderate (++), strong (+++) staining. **B.** The representative immunoreactivity of paired tumor and normal tissues in buccal mucosal, tongue, and lip subsites.

First, the intensity score of the DDX60 staining was measured using a numerical scale (0, no expression; 1, weak expression; 2, moderate expression; and 3, strong expression; Figure 1A). The immunoreactivity of DDX60 was higher in tumor tissues compared to that in CTAN tissues at the buccal mucosa, tongue, and lip subsites (Figure 1B). Moreover, DDX60 was highly expressed in 384 OSCC tissues (p<0.001), including 136 BMSCC tissues (p= 0.007), 192 TSCC tissues (p<0.001), and 56 LSCC tissues (p<0.001) compared to its expression in CTAN tissues (Table 1). These results indicated that DDX60 may be involved in the tumorigenesis of OSCC. Furthermore, DDX60 expression in CTAN (p<0.001) or tumor tissues (p= 0.049) was significantly different between the buccal mucosa, tongue, and lip subsites (Table 1). The post hoc analysis of the CTAN tissues revealed that the expression level of DDX60 was significantly higher in the buccal mucosal epithelium than in the lip epithelium, and its expression was also significantly higher in the tongue epithelium than in the lip epithelium. Additional post hoc analyses of the tumor tissues showed that the expression level of DDX60 was also significantly higher in buccal mucosal SCC than in lip SCC. Altogether, these results revealed that DDX60 expression may be correlated with tumorigenesis in OSCC, and its expression was quite different between the three different subsites of the oral cavity. Thus, a stratification analysis according to the three different subsites was performed to evaluate the effect of DDX60 expression on tumorigenesis, clinicopathologic outcomes, and survival.

**Table 1:** The comparisons of DDX60 expression between corresponding tumor adjacent normal and oral SCC tissues and between different subsites of oral SCC

Variables	No.	Tumor adjacent normal		Tumor		Z	*p*-value*
		Mean±SD	Median	Mean±SD	Median		
*Total:* Oral SCC	384	3.62±1.63	4.00	4.24±1.12	4.00	6.189	**<0.001**
*Subsites:*							
Buccal mucosal SCC	136	4.01±1.51^a^	4.00	4.43±1.01^c^	5.00	2.712	**0.007**
Tongue SCC	192	3.66±1.55^b^	4.00	4.18±1.19	4.00	3.663	**<0.001**
Lip SCC	56	2.54±1.74^a^^b^	2.50	4.02±1.09^c^	4.00	4.658	**<0.001**
		χ^2^=28.768; ***p* <0.001**^†^		χ^2^=6.036; ***p***=0.049**^†^**			

Abbreviations: SCC, squamous cell carcinoma; SD, standard deviation.

* p-values were the comparisons of DDX60 between tumor adjacent normal and tumor; they were estimated by Wilcoxon matched-pairs signed-ranks test.

† *p*-*values were the comparisons of DDX60 between three different subsites; they were estimated by Kruskal-Wallis one-way ANOVA test. Post-hoc test was estimated by*

a p<0.001;

b p<0.001;

c p=0.026.

Bold values denote statistically significant.

### Correlation between DDX60 expression and tumorigenesis of OSCC patients according to the status of cigarette smoking, BQ chewing, and alcohol drinking

Cigarette smoking, BQ chewing, and alcohol drinking are well-known risk factors for OSCC. After stratification of the status of BQ chewing, cigarette smoking, and alcohol drinking, the expression levels of DDX60 between tumor and tumor adjacent normal tissues were compared. The results showed that DDX60 expression is significantly associated with tumor development in TSCC patients with BQ chewing (p<0.001), smoking (p<0.001), or alcohol drinking (p<0.001) compared to the expression in TSCC patients without BQ chewing, cigarette smoking, or alcohol drinking (Supplementary Tables 1, 2, 3). Additionally, DDX60 expression is correlated with tumorigenesis in LSCC patients with or without BQ chewing, smoking, and alcohol drinking (Supplementary Tables 1, 2, and 3). However, DDX60 expression is not correlated to tumorigenesis in BMSCC patients with or without BQ chewing, cigarette smoking, and alcohol drinking (Supplementary Tables 1, 2, 3). Therefore, DDX60 was independently correlated with the tumorigenesis of OSCC without any interactions of BQ chewing, smoking, and drinking, except for TSCC.

### DDX60 expression and clinicopathological outcomes in patients with OSCC and the three primary subsites of OSCC

We further studied the association between DDX60 expression and clinicopathologic parameters, including sex, age, cell differentiation, pathological stage, T classification and N classification. As shown in Table 2, DDX60 expression was positively associated with advanced pathological stage (III, IV, p= 0.042) and large tumor size (T3-T4, p= 0.032) in BMSCC patients, whereas a higher level of expression was correlated with sex in the male gender (p= 0.023), lower grade (such as well and moderate) cell differentiation (p< 0.001) and large tumor size (T3-T4, p= 0.017) in TSCC patients. Overall, a higher DDX60 expression was observed in OSCC patients with male gender (p= 0.002), low-grade cell differentiation (p= 0.004), advanced pathological stage (p= 0.023), and large tumor size (T3-T4, p= 0.001). However, DDX60 expression was not correlated with any clinicopathologic parameters in LSCC. These findings indicated that DDX60 may play an important but different role in the clinicopathologic outcomes of OSCC according to the various subsites.

**Table 2:** Expression of DDX60 and clinicopathologic outcomes in patients with oral SCC and three primary subsites

Variable	Buccal mucosal SCC (n=180)				Tongue SCC (n=241)				Lip SCC (n=73)				Oral SCC (n=494)			
	%	Mean±SD	Median	*p*-value	%	Mean±SD	Median	*p*-value	%	Mean±SD	Median	*p*-value	%	Mean±SD	Median	*p*-value
Sex																
Female	2.2	4.50±1.29	4.50	0.792*	12.0	3.69±1.07	4.00	0.023*	9.6	3.29±1.70	3.00	0.098^†^	8.1	3.70±1.22	4.00	0.002*
Male	97.8	4.36±1.06	4.00		88.0	4.22±1.18	4.00		90.4	4.26±0.93	4.00		91.9	4.28±1.10	4.00	
Age, y																
≦50	44.4	4.33±0.96	4.00	0.684*	51.0	4.20±1.23	4.00	0.504*	21.9	4.25±1.00	4.00	0.716*	43.1	4.28±1.09	4.00	0.379*
>50	55.6	4.39±1.14	5.00		49.0	4.10±1.12	4.00		78.1	4.14±1.08	4.00		56.9	4.19±1.14	4.00	
Subsite																
Buccal	100.0	4.36±1.06	4.00	-	-	-	-	-	-	-	-	-	36.4	4.36±1.06	4.00	0.146^‡^
Tongue	-	-	-		100.0	4.15±1.17	4.00		-	-	-		48.8	4.15±1.17	4.00	
Lip	-	-	-		-	-	-		100.0	4.16±1.05	4.00		14.8	4.16±1.05	4.00	
Cell differentiation																
Well	26.1	4.45±0.93	5.00	0.663^‡^	10.8	4.65±0.89^a^^b^	5.00	**<0.001^§^**	47.9	4.37±0.77	4.00	0.229^‡^	21.9	4.47±0.87^d^^e^	4.50	**0.004^§^**
Moderate	68.9	4.35±1.08	4.00		82.2	4.20±1.08^a^^c^	4.00		47.9	3.94±1.26	4.00		72.3	4.22±1.10^d^^f^	4.00	
Poor	5.0	4.11±1.45	5.00		7.1	2.88±1.73^b^^c^	3.00		4.1	4.33±1.15	5.00		5.9	3.41±1.68^e^^f^	3.00	
AJCC pathological stage																
I, II	61.7	4.23±1.10	4.00	0.042*	68.9	4.10±1.21	4.00	0.316*	79.5	4.14±1.05	4.00	0.676*	67.8	4.15±1.14	4.00	0.023*
III, IV	38.3	4.57±0.98	5.00		31.1	4.27±1.09	4.00		20.5	4.27±1.10	5.00		32.2	4.40±1.05	5.00	
T classification																
T1, T2	75.6	4.26±1.08	4.00	0.032*	79.7	4.06±1.19	4.00	0.017*	82.2	4.13±1.05	4.00	0.592*	78.5	4.14±1.13	4.00	0.001*
T3, T4	24.4	4.66±0.94	5.00		20.3	4.51±1.06	4.00		17.8	4.31±1.11	5.00		21.5	4.55±1.02	5.00	
N classification																
N0	75.6	4.34±1.08	4.00	0.612*	80.1	4.17±1.18	4.00	0.746*	94.5	4.19±1.03	4.00	0.423*	80.6	4.23±1.12	4.00	0.932*
N1, N2	24.4	4.43±1.02	5.00		19.9	4.10±1.15	4.00		5.5	3.75±1.50	4.00		19.4	4.24±1.11	4.00	

Abbreviations: SCC, squamous cell carcinoma; AJCC, American Joint Committee on Cancer.

* p values were estimated by student's t-test.

† p values was estimated by Mann-Whitney U test.

‡ p values were estimated by one-way ANOVA test.

§ p values were estimated by Kruskal-Wallis one-way ANOVA test.

a p=0.041;

b p<0.001;

c p=0.001;

d p=0.042;

e p=0.002;

f p=0.015.

Bold values denote statistically significant.

### DDX60 expression and survival of OSCC patients

**Figure 2:**
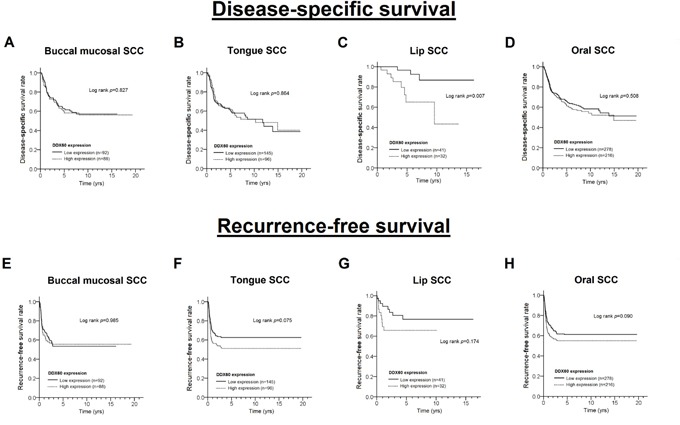
The Kaplan-Meier curves for disease-specific survival and recurrence-free survival with different levels of DDX60 expression in patients with BMSCC (A, E), TSCC (B, F), LSCC (C, G) and OSCC (D, H)

To determine whether DDX60 is involved in the survival of OSCC, a log rank test (Figure 2A-2H) and Cox proportional hazards models (Table 3) were used. The results showed that high DDX60 expression was not associated with a poor DSS in OSCC patients, except in LSCC patients (log rank test: p= 0.007, Figure 2C; Adjusted Hazard Ratio (AHR) = 5.13; 95% Confidence Interval (CI)= 1.32-19.90; p= 0.018, Table 3). In addition, OSCC patients with a high DDX60 expression level showed a shorter recurrence-free survival (RFS) (log rank test: p= 0.090, Figure 2H; AHR= 1.36; 95% CI= 1.02-1.81; p= 0.034, Table 3), particularly TSCC patients (log rank test: p= 0.075, Figure 2F; AHR= 1.57; 95% CI= 1.05-2.35; p= 0.027, Table 3).

**Table 3:** The expression levels of DDX60 and disease-specific and recurrence-free survival for patients of oral SCC and three primary subsites

DDX60 (50th percentile)		No. (%)	Disease-specific survival		Recurrence-free survival	
			AHR (95% CI)	*p*-value*	AHR (95% CI)	*p*-value*
Buccal mucosal SCC	Low (0-4)	92 (51.1)	1.00		1.00	
	High (5-7)	88 (48.9)	0.90 (0.57-1.42)	0.638	0.90 (0.57-1.42)	0.638
Tongue SCC	Low (0-4)	145 (60.2)	1.00		1.00	
	High (5-7)	96 (39.8)	1.04 (0.70-1.54)	0.844	1.57 (1.05-2.35)	**0.027**
Lip SCC	Low (0-4)	41 (56.2)	1.00		1.00	
	High (5-7)	32 (43.8)	5.13 (1.32-19.90)	**0.018**	2.58 (0.98-6.83)	0.056
Total: Oral SCC	Low (0-4)	278 (56.3)	1.00		1.00	
	High (5-7)	216 (43.7)	1.07 (0.80-1.42)	0.649	1.36 (1.02-1.81)	**0.034**

*Abbreviations: SCC, squamous cell carcinoma; CHR, crude hazard ratio; CI, confidence interval; AHR, adjusted hazard ratio*.

* p-value were adjusted for cell differentiation (moderate + poor vs. well) and AJCC pathological stage (stage III+IV vs. stage I+II) by multiple Cox‘s regression.

Bold values denote statistically significant.

**Figure 3:**
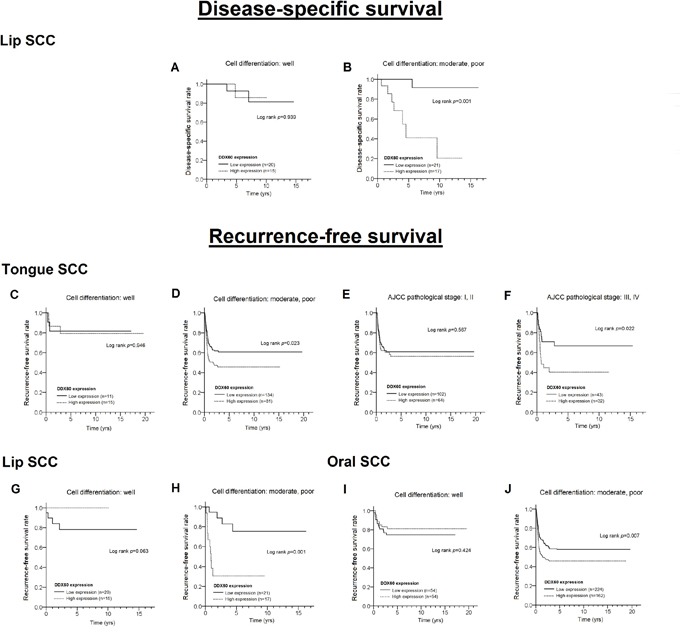
Differences in the survival curves between patients with high and low levels of DDX60 expression in LSCC (A, B for DSS; G, H for RFS), TSCC (C-F for RFS), and OSCC (I, J for RFS), stratified according to cell differentiation and the pathological stage

We further stratified BMSCC, TSCC, LSCC, or OSCC patients into two groups based on clinicopathologic factors, such as gender (male group and female group), age (≤ 50 and > 50), cell differentiation (well and moderate/poor), AJCC pathological stage (I+II and III+IV), T stage (T1+T2 and T3+T4), N stage (N0 and N1+N2), and receiving postoperative radiotherapy (Ever and Never), to assess the effect of the DDX60 expression level (high vs. low) on DSS and RFS (Figure 3A-3J). For LSCC patients, a high level of DDX60 expression was significantly associated with poor DSS in individuals with moderate or poor cell differentiation (Figure 3B, log rank test: p= 0.001; Cox's regression after adjustment of pathological stage: AHR= 15.19, 95% CI= 1.81 - 127.322, p= 0.012, data not shown). For TSCC patients, a high level of DDX60 expression was associated with poor RFS in individuals with moderate or poor cell differentiation (Figure 3D, log rank test, p= 0.023; Cox's regression after adjustment of pathological stage: AHR= 1.60, 95% CI= 1.06 - 2.41, p = 0.025; data not shown) and those with advanced pathological stage (Figure 3F, log rank test: p= 0.022; Cox's regression after adjustment of cell differentiation: AHR= 2.53, 95% CI= 1.23 - 5.18, p= 0.011, data not shown). Additionally, high DDX60 expression was a significant predictor of short RFS only in LSCC patients with moderate or poor cell differentiation (Figure 3H, log rank test: p= 0.001; Cox's regression after adjustment of pathological stage: AHR= 6.88, 95% CI: 2.01-23.57, p= 0.002, data not shown). For OSCC patients, high levels of DDX60 were correlated with shorter RFS in individuals with moderate or poor cell differentiation (Figure 3J, log rank test: p= 0.007; Cox's regression after adjustment of pathological stage: AHR: 1.48, 95% CI: 1.09-1.99, p= 0.011, data not shown). Thus, DDX60 is a new independent negative prognostic biomarker of oral cancer, particularly for TSCC and LSCC. In addition, the effect of DDX60 expression on survival was inconsistent between the three OSCC subsites.

## DISCUSSION

Virus infections by human papillomavirus, herpes simplex virus, and Epstein-Barr virus have been associated with an increased risk of OSCC [25, 26]. DDX60 is a type I interferon-inducible gene in response to viral infections [12]. Conversely, DDX60 can induce type I IFN (IFN-α/β), which is a well-known potent cytokine under an antiviral innate immune response [27]. Surprisingly, type I IFN has been implicated in cancer development [28, 29]. For example, IFN-β enhances the motility of oral cancer cells by stimulating ubiquitin cross-reactive protein (UCRP) expression [30]. Moreover, DDX60 is a member of the DEAD box RNA helicase protein family, which is involved in most cellular processes essential for cancer development, such as cell proliferation [2]. Indeed, several overexpressed DDX proteins are associated to advanced clinical stage, poor survival, and early recurrence in human cancers [2, 3, 31, 32], indicating their important effects in cancer development and progression. Herein, we demonstrated the association of DDX60 with tumorigenesis and the clinicopathologic significance of OSCC and characterized the potential of this protein as a prognostic biomarker of OSCC, particularly in the recurrence of TSCC and survival of LSCC.

In our study, the increased expression of DDX60 was correlated with the development of OSCC, including the three subsites BSCC, TSCC, and LSCC. Many DDX proteins have been reported to participate in tumorigenesis by regulating cell proliferation [2, 33, 34]. For example, DDX5 promotes the proliferation of non-small-cell lung cancer cells for tumorigenesis by directly interacting with β-catenin to promote the transcription of cyclin D1 and c-Myc [32]. DDX21 promotes c-Jun activity by EGF signaling in the tumorigenesis of breast cancer [35]. DDX23 promotes the proliferation of glioma cells by modulating miR-21 biogenesis [36]. DDX5 promotes glioma cell proliferation and tumor growth through the direct regulation of the NF-kB transcription factor p50 [37]. Consistently, DDX60 expression was increased in tumor tissues and positively associated with larger tumor sizes in OSCC patients, particularly for TSCC and BMSCC, indicating that DDX60 might induce cell proliferation through the verified mechanisms of DDX proteins in tumorigenesis. Moreover, oral cancer cells silenced using siRNA against DDX60 also showed reduced cell growth (Supplementary Figure 1). Therefore, DDX60 might involve in cell proliferation for tumorigenesis in our studied OSCC patients.

Our results also indicated that DDX60 is significantly associated with early recurrence in OSCC, particularly in TSCC (Table 3). Accordingly, cancer stem cell-like markers [38] and epithelial-mesenchymal transition (EMT) biomarkers (vimentin up-regulation and E-cadherin and β-catenin down-regulation) are associated with recurrence of OSCC patients [39]. It has been reported that DDX4 colocalizes with the cancer stem cell marker CD133 in ovarian cancers [40]. The finding of DDX5 in promoting EMT through facilitating β-catenin nuclear translocation has been reported [41]. Moreover, DDX3X induced the phenotype of EMT by switching from E-cadherin to N-cadherin [42]. Thus, DDX60 might also be involved in recurrence by regulating the EMT or cancer stem cell-like biomarkers in OSCC and TSCC, which should be further investigated.

In the stratification analysis in this study, earlier recurrence and death were observed in patients with progressive diseases (Figure 3), such as moderately or poorly differentiated tumor (LSCC, TSCC, and OSCC) and advanced stage diseases (only in TSCC). Based on the adjusted hazards ratio values or survival curves (Table 3 and Figures 2-3), we found that the effect of DDX60 on recurrence or survival was mild (Figure 2) and only obvious for progressive diseases (Figure 3). In patients with progressive diseases, immune system impairment results in the enhanced motility of oral cancer and increased potential for early recurrence or death, which may result from DDX60-induced IFN-β. Interestingly, our results showed that the increased expression of DDX60 was correlated with well-differentiated TSCC and OSCC (Table 2), which seems illogical in cancer. However, the results of the stratified analyses according to the three different subsites revealed that the correlation of DDX60 expression between cell differentiation, tumor stage, and survival in each subsite is logical (Tables 2 and 3).

In this study, our data indicated different DDX60 expression levels in the tumor adjacent normal tissues (p<0.001) or tumor tissues (p= 0.049) between the buccal, tongue, and lip subsites (Table 1). Furthermore, BQ chewing, cigarette smoking, and alcohol drinking might not confound the effect of DDX60 on oral cancer development for OSCC, except for TSCC. Moreover, different clinicopathologic outcomes (Table 2) at the three different subsites were also observed. Indeed, the high expression of DDX60 was associated with early recurrence in TSCC and OSCC and death in LSCC (Table 3). Therefore, our data suggest that the effect of DDX60, a DEAD box protein, on tumorigenesis and the prognosis of oral cancer is subsite-specific, which is consistent with a previous study [43].

HPV infection is a high risk and poor prognostic factor for OSCC, particularly for male patients [44, 45] and patients with poor-differentiated carcinoma in the head and neck [46]. DDX60 expression is induced after virus infections. Nevertheless, our preliminary data showed that DDX60 expression was not correlated with the HPV biomarker p16 expression, which was assayed according to the method proposed by Liang et al. [47] (neither nuclear expression, correlation coefficient: -0.063, p= 0.331; nor cytoplasm expression, correlation coefficient: 0.039, p= 0.542; data not shown). Therefore, DDX60 might play an HPV-independent role in the tumorigenesis and prognosis our OSCC patients. The relationship between DDX60 expression and other different oncogenic virus infections in OSCC patients will need to be further investigated, particularly for male patients and individuals with well-differentiated OSCC.

Moreover, the activation of inflammatory cytokines [ex. type I interferon (IFN-α/β), tumor necrosis factor-alpha (TNF-α), and type II interferon (IFN)-γ] in response to viral/chemical carcinogens is significantly associated with OSCC [27, 29, 48]. DDX60 could be induced by type I interferon, and the possibility that DDX60 could be induced by other inflammatory cytokines could not be excluded [12, 14] and needs further verification.

Notably, there are some limitations in the present study as follows: (1) there are 70% sequence identities between DDX60 and DDX60L [14], implying that the anti-DDX60 antibody used in the study may cross react with DDX60L. To exclude the potential effect of DDX60L on tumorigenesis and prognosis in OSCC, IHC staining and statistical analyses are needed. (2) The sample size of lip cancer patients might not be large enough. The risk of recurrence is high (AHR= 2.58, 95% CI: 0.98-6.83) but with a borderline level of statistical significance (p= 0.056) because of the small statistical power. (3) There are 32% (n= 122), 28% (n= 106), and 28% (n= 109) of OSCC patients without BQ chewing, smoking, and drinking data, respectively, in the chart (Supplementary Tables 1-3). Therefore, the interactions between BQ chewing, cigarette smoking, or alcohol drinking and DDX60 expression on tumorigenesis need further validation in a prospective study using a questionnaire survey.

In conclusion, increased DDX60 expression was involved in not only the tumorigenesis but also the unfavorable prognosis of OSCC, particularly in the tongue and lip subsites.

## MATERIALS AND METHODS

### Patients and tissue subjects

The margin-free (margin-size ≥ 0.2 cm) specimens of 494 OSCC tissues, including BMSCC (n= 180), TSCC (n= 241), LSCC (n= 73), and 384 corresponding CTAN tissues (for BMSCC, n= 136; for TSCC, n= 192; for LSCC, n= 56) were obtained from the Department of Pathology, Kaohsiung Veterans General Hospital between 1990 and 2013. The survival time was estimated from the time of operation to November 2013. Pathological stage and TNM classification were determined at the time of the initial resection of the tumor in accordance with the guidelines of the 2002 American Joint Committee on Cancer (AJCC) system. The protocol for this study was approved by the Institutional Review Board at Kaohsiung Veterans General Hospital (IRB number: VGHKS14-CT6-18).

### Tissue microarray construction

A tissue microarray block contained 149 cores, 1.5 mm in diameter, including 48 trios. Each trio contained two cores from the tumor tissue and one core from the CTAN of the same patient. Five cores of normal uvula epithelium from other individuals were also included in each TMA block [49]. The representative area of tumor and non-cancer epithelium tissues was selected by a senior oral cancer pathologist from hematoxylin-eosin-stained sections for coring cylindrical tissues from paraffin-embedded tissues. In addition, not all histological contents of the tumor cores and CTAN cores were correct. Those cores with incorrect contents were excluded. Therefore, a total of 988 tumor cores (494 / 509 patients, 97.1%) and 384 CTAN cores (384 / 433 patients, 88.7%) were included in this study.

### Immunohistochemistry (IHC)

TMA blocks were cut into 4-μm paraffin sections, dewaxed in xylene, rehydrated through a series of graded alcohols, and subsequently washed for 5 minutes with phosphate-buffered saline (PBS) [50]. The Novolink max polymer detection system (Leica, Newcastle Upon Tyne, United Kingdom) was used for the following immunostaining processes. Antigen retrieval was performed by immersion in Tris-EDTA (10 mM, pH 9.0) for 10 minutes at 125°C in a pressure boiler. Endogenous peroxidase activity was blocked at room temperature for 30 minutes with 3% hydrogen peroxide in methanol. After blocking, the slides were incubated overnight at 4°C in a wet chamber with the goat anti-DDX60 (C-18) [sc-242561] monoclonal antibody (dilution 1:200; Santa Cruz Biotechnology, Dallas, Texas, USA) in primary antibody diluent (ScyTek Laboratories, Logan, Utah, USA). The color was developed using a 0.03% diaminobenzidine solution for 2 minutes at room temperature, and subsequently the sections were counterstained with hematoxylin. Positive controls were obtained from colon adenocarcinoma sections. The substitution of the primary antibody with antibody dilution buffer served as a negative control.

### IHC analysis and scoring

Two senior pathology technicians (Miss Huei-Cin Sie and Huei-Han Liu) blinded to the clinical data used a semi-quantitative approach to grade DDX60 immunoreactivity. Initially, an oral cancer pathologist (Dr. Ting-Ying Fu) accompanied these two technicians to evaluate slides until all the discrepancies were resolved. Subsequently, both technicians independently reviewed the slides, except the cores with incorrect or uncertain contents, which must be scored by the pathologist. In addition, any disagreement of IHC scores between these two technicians was re-evaluated by the pathologist. Then, only one technician (Miss Sie) reviewed all slides, and 5% of the core samples from each intensity in her reviews were randomly selected for re-evaluation by the pathologist. If the pathologist and technician disagreement regarding the IHC scores was > 5%, the pathologist accompanied the technician to re-evaluate the slides until all the discrepancies were resolved. Then, this technician re-evaluated and re-scored all slides until another random core sample (5%) resulted in a scoring agreement ≧ 95%. Approximately 36% of the core samples were scored or re-evaluated by a pathologist. In the final evaluation, the pathologist and technician agreement of the DDX60 score was 97.59% in this study.

The percentage of cell staining at each intensity level was graded as 0 (<5%), 1 (5~25%), 2 (26~50%), 3 (51-75%), and 4 (>75%). The intensity score and percentage of the positive cells were added to produce the final scores (0-7). For the survival analysis, the expression level was dichotomized as low and high expression using a cutoff set at the 50^th^ percentile based on the distribution of the DDX60 score. The cutoff value was 5.

### Statistical analysis

The SPSS software program (version 20.0, SPSS Inc., Chicago, IL, USA) was used for all statistical analyses. Differences in DDX60 expression between the paired tissues (tumor vs. CTAN) were evaluated using the Wilcoxon matched-pairs signed-ranks test. The Kruskal-Wallis one-way ANOVA test, one-way ANOVA test, Mann-Whitney U test, or Student's t-test were used to evaluate the relationship between the protein expression levels and the clinicopathologic outcomes. Disease-specific survival was measured from the time of the initial resection of the primary tumor to the date of the cancer-specific death or the last follow-up. RFS was calculated from the date of the initial resection of the primary tumor to the date of recurrence or the last follow-up. Moreover, RFS survival includes both local and regional RFS. The cumulative survival curves were estimated using the Kaplan-Meier method. The survival curves were compared using a log-rank test. A Cox proportional hazards model was used to determine the independent predictors of survival using factors significant on a univariate analysis as covariates. p-values < 0.05 were considered statistically significant.

## SUPPLEMENTARY MATERIALS FIGURE AND TABLES









## Abbreviations

AHR: Adjusted Hazard Ratio
AJCC: American Joint Committee on Cancer
BMSCC: Buccal mucosal SCC
CI: Confidence Interval
CTAN: Corresponding tumor adjacent normal
DDX: DEAD-box
DSS: Disease-specific survival
IFN: Interferon
IHC: Immunohistochemistry
OSCC: Oral squamous cell carcinoma
LSCC: Lip SCC
PBS: phosphate-buffered saline
RFS: Recurrence-free survival
TSCC: Tongue SCC
UCRP: Ubiquitin cross-reactive protein
