# Supplementary material for: Subsite-specific association of DEAD box RNA helicase DDX60 with the development and prognosis of oral squamous cell carcinoma
Source: Oncotarget. 2016 Nov 8;7(51):85097–108. doi: 10.18632/oncotarget.13197 (PMC5356722; doi:10.18632/oncotarget.13197)
Supplement: Supplementary file 1 [file oncotarget-07-85097-s001.pdf]

Subsite-specific association of DEAD box RNA helicase DDX60 with the development and prognosis of oral squamous cell carcinoma

SUPPLEMENTARY FIGURE AND TABLES

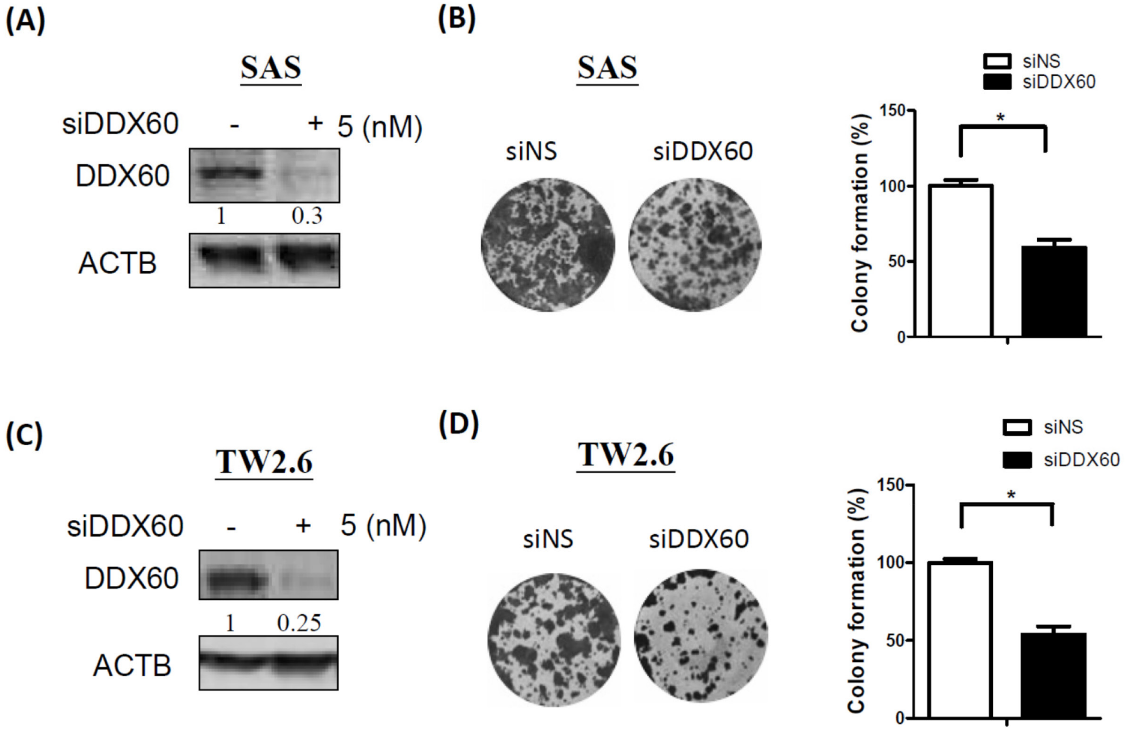

Supplementary Figure 1: Silencing DDX60 reduced cell growth in human oral cancer cells. Human oral cancer SAS **A, B.** or TW2.6 **C, D.** cells were transiently transfected with 5 nM scrambled siRNA or siRNA against DDX60 for 72 h. (A) The cells were lysed for immunoblotting to determine the protein levels of DDX60 and ACTB. (B) Cell proliferation was measured by clonogenic assays. The results are expressed as the mean ± SEM from three individual experiments.

Supplementary Table 1: The comparisons of DDX60 expression between corresponding tumor adjacent normal and oral SCC by different status of BQ chewing and different subsites of oral SCC

See Supplementary File S1.

Supplementary Table 2: The comparisons of DDX60 expression between corresponding tumor adjacent normal and oral SCC by different status of smoking and different subsites of oral SCC

See Supplementary File S2.

Supplementary Table 3: The comparisons of DDX60 expression between corresponding tumor adjacent normal and oral SCC by different status of drinking and different subsites of oral SCC

See Supplementary File S3.
