# Supplementary material for: Subsite-specific association of DEAD box RNA helicase DDX60 with the development and prognosis of oral squamous cell carcinoma
Source: Oncotarget. 2016 Nov 8;7(51):85097–108. doi: 10.18632/oncotarget.13197 (PMC5356722; doi:10.18632/oncotarget.13197)
Supplement: Supplementary file 2 [file oncotarget-07-85097-s002.doc]

| **Supplementary Table 1.** The comparisons of DDX60 expression between corresponding tumor adjacent normal and oral SCC by different status of BQ chewing and different subsites of oral SCC | | | | | | | | | |
| --- | --- | --- | --- | --- | --- | --- | --- | --- | --- |
| Variables | BQ chewing^＊^ | No. | Tumor adjacent normal | |  | Tumor | | Z | *p*-value^†^ |
|  |  |  | Mean±SD | Median |  | Mean±SD | Median |  |  |
| Buccal mucosal SCC | No | 26 | 4.35±1.32 | 4.00 |  | 4.62±1.10 | 5.00 | 1.194 | 0.233 |
|  | Yes | 38 | 4.24±1.48 | 4.00 |  | 4.50±0.95 | 4.00 | 0.750 | 0.454 |
|  |  |  |  |  |  |  |  |  |  |
| Tongue SCC | No | 55 | 3.85±1.63 | 4.00 |  | 4.09±1.38 | 4.00 | 0.952 | 0.341 |
|  | Yes | 87 | 3.45±1.45 | 4.00 |  | 4.39±1.06 | 4.00 | 4.552 | **<0.001** |
|  |  |  |  |  |  |  |  |  |  |
| Lip SCC | No | 15 | 1.80±1.93 | 2.00 |  | 3.80±1.32 | 4.00 | 2.527 | **0.012** |
|  | Yes | 41 | 2.80±1.60 | 3.00 |  | 4.10±1.00 | 4.00 | 3.944 | **<0.001** |
|  |  |  |  |  |  |  |  |  |  |
| Total:  Oral SCC | No | 96 | 3.67±1.79 | 4.00 |  | 4.19±1.32 | 4.00 | 2.512 | **0.012** |
|  | Yes | 166 | 3.47±1.57 | 4.00 |  | 4.34±1.03 | 4.00 | 5.896 | **<0.001** |
| *Abbreviations: SCC, squamous cell carcinoma; SD, standard deviation.*  ^＊^There were 72 BSCC patients, 50 TSCC patients, and 122 OSCC patients were excluded in the stratification analysis because of no BQ data in their medical chart.  ^†^*p-values* *were estimated by Wilcoxon matched-pairs signed-ranks test.*  Bold values denote statistically significant. | | | | | | | | | |
