# Supplementary material for: Subsite-specific association of DEAD box RNA helicase DDX60 with the development and prognosis of oral squamous cell carcinoma
Source: Oncotarget. 2016 Nov 8;7(51):85097–108. doi: 10.18632/oncotarget.13197 (PMC5356722; doi:10.18632/oncotarget.13197)
Supplement: Supplementary file 4 [file oncotarget-07-85097-s004.doc]

| **Supplementary Table 3.** The comparisons of DDX60 expression between corresponding tumor adjacent normal and oral SCC by different status of drinking and different subsites of oral SCC | | | | | | | | | |
| --- | --- | --- | --- | --- | --- | --- | --- | --- | --- |
| Variables | Drinking^＊^ | No. | Tumor adjacent normal | |  | Tumor | | Z | *p*-value^†^ |
|  |  |  | Mean±SD | Median |  | Mean±SD | Median |  |  |
| Buccal mucosal SCC | No | 29 | 4.17±1.44 | 4.00 |  | 4.59±1.02 | 5.00 | 1.624 | 0.104 |
|  | Yes | 46 | 4.24±1.49 | 4.00 |  | 4.33±1.08 | 4.00 | 0.061 | 0.952 |
|  |  |  |  |  |  |  |  |  |  |
| Tongue SCC | No | 51 | 3.80±1.61 | 4.00 |  | 4.04±1.33 | 4.00 | 1.023 | 0.306 |
|  | Yes | 93 | 3.52±1.48 | 4.00 |  | 4.42±1.09 | 4.00 | 4.408 | **<0.001** |
|  |  |  |  |  |  |  |  |  |  |
| Lip SCC | No | 19 | 2.16±1.77 | 2.00 |  | 3.68±1.06 | 3.00 | 2.718 | **0.007** |
|  | Yes | 37 | 2.73±1.71 | 3.00 |  | 4.19±1.08 | 4.00 | 3.728 | **<0.001** |
|  |  |  |  |  |  |  |  |  |  |
| Total:  Oral SCC | No | 99 | 3.60±1.74 | 4.00 |  | 4.13±1.23 | 4.00 | 2.891 | **0.004** |
|  | Yes | 176 | 3.54±1.61 | 4.00 |  | 4.35±1.08 | 4.00 | 5.377 | **<0.001** |
| *Abbreviations: SCC, squamous cell carcinoma; SD, standard deviation.*  ^＊^There were 61 buccal mucosal SCC patients, 48 tongue SCC patients, and 109 oral SCC patients were excluded in the stratification analysis because of no drinking data in their medical chart.  ^†^*p-values* *were estimated by Wilcoxon matched-pairs signed-ranks test.*  Bold values denote statistically significant. | | | | | | | | | |
